# Healthy Minds for Healthy Hearts: Tackling Stress-Induced Cardiac Events During the FIFA World Cup 2022

**DOI:** 10.2147/VHRM.S390549

**Published:** 2022-12-06

**Authors:** Muna Abed Alah, Sami Abdeen, Nagah Selim

**Affiliations:** 1 Community Medicine Department, Hamad Medical Corporation (HMC), Doha, Qatar; 2 Community Medicine Department, Primary Health Care Corporation, Doha, Qatar; 3 Public health and Preventive medicine department, Cairo University, Cairo, Egypt

**Keywords:** FIFA World Cup, soccer, football, stress, cardiac, mental

## Abstract

Millions of people are looking forward to the biggest event this year “FIFA World Cup 2022” taking place in the state of Qatar. This event is an opportunity for people around the world to socialize, connect, celebrate, and enjoy watching football matches. However, the emotional stress experienced by football players and fans during a such major sport event can sometimes result in unfavorable physiological responses that can adversely affect the heart leading to adverse cardiac consequences. In this mini-review, we summarized the evidence and pathophysiology of stress-induced cardiac events during football games, and the potential strategies to prevent stress-induced cardiac events during the FIFA World Cup 2022.

## Introduction

As Qatar hosts the FIFA World Cup 2022 between November and December this year, the event presents an opportunity to promote physical activity and sport worldwide, especially by scaling up the Generation Amazing program that was first established during Qatar’s bid to host the FIFA World Cup 2022^1^. This program utilizes “a football for development approach” which is a type of football training on and off the pitch that reinforces principles like gender equality and inclusivity, and life skills like communication, organization, teamwork, and leadership.^2^ The FIFA World Cup 2022 is an opportunity to encourage other healthy lifestyle habits and behaviors by spreading health-promoting messages through different awareness campaigns. Adopting healthy behaviors is of paramount importance to the prevention and control of non-communicable diseases most importantly cardiovascular diseases (CVD), Diabetes Mellitus (DM), and hypertension (HTN) which are interrelated.^3^ Maintaining emotional well-being and the ability to cope with stress are integral components of lifestyle medicine which is an evolving field of medicine that utilizes an evidence-based approach for treating, preventing, and even reversing chronic diseases by replacing unhealthy behaviors with healthy ones.^4^ It is established in the literature that long-term exposure to stress can precipitate coronary heart disease, while acute stress can trigger the occurrence of cardiac events.^5–7^ Moreover, stress can also increase the prevalence and severity of several CVD risk factors, including HTN, DM, and obesity.^6^ The adverse health consequences of stressful conditions depend on the physiological responses to different stress exposures that vary from one person to another.^7^ Both football players and fans experience a great deal of stress, particularly during important sporting events.^8^ In this mini-review, we summarized the evidence and pathophysiology of stress-induced cardiac events during football games, the strategies in place to promote healthy lifestyles, and other potential strategies needed to prevent stress stress-induced cardiac events during major sporting events like the FIFA World Cup 2022.

### Evidence of Stress-Induced Cardiac Events During Major Sporting Events with a Particular Focus on Football Games

During major competitions, football players face a series of stressors of variable intensities that can influence their physical performance and health during the match.^9^ Several sources of stress for football players have been spotted in the literature such as the training environment, competition, fear of injury, goals, and expectations, coaching styles, team atmosphere, and media stress.^9–12^ The relationship between the stress experienced by people while watching football games and the risk of cardiovascular events is controversial. Recent systematic reviews and meta-analyses showed an increased risk of fatal and non-fatal cardiovascular events in both men and women while watching football matches.^13^,^14^ A study conducted during the FIFA World Cup 2006 in Germany found that viewing a stressful football match more than doubles the risk of an acute cardiovascular event.^15^ During England’s 1998 World Cup, there was an increase of 25% in the admissions for acute myocardial infarction on the day England lost to Argentina in a penalty shoot-out and the following two days, while such an increase was not observed for other diagnoses or on the days of the other England matches.^16^ About 14 excess cardiovascular deaths occurred among men on the day of the match when the Dutch football team was eliminated from the European football championship in 1996.^17^ Hospital admissions due to myocardial infarction increased during the 2014 FIFA World Cup in Germany.^18^ A recently published study that assessed the association between emotional stress and acute coronary syndrome during the Spanish league competition reported a 30% increase in the number of admissions due to acute coronary syndrome among high-risk men on the days of the loss of the local team.^19^ Just recently, on September 10, 2022, a Cadiz fan had a cardiac arrest at the Stadium during the 4–0 defeat to Barcelona, he was resuscitated before being admitted to the intensive care unit of a local hospital.^20^ Takotsubo cardiomyopathy or what is known as “broken heart syndrome” or stress-induced cardiomyopathy has been reported among fans after the defeat of their favorite teams^21^,^22^ (Table 1).

**Table 1:** Summary of the Evidence of Stress-Induced Cardiac Events During Major Football Tournaments

Studies (Author, Year, Reference Number)	Related Sport Events	Main Findings
Lin et al, 2019^13^	A systematic review and metanalysis of studies assessing cardiovascular events during Football matches which included the FIFA World Cup, European Football Championship, English Football Tournament, and Australian Football between 1996 and 2010	Viewing football matches was associated with a higher risk of fatal overall CVD (RR: 1.06, 95% CI: 1.01–1.12), and a higher risk of non-fatal myocardial infarction (RR: 1.20, 95% CI: 1.04–1.38) in both men and women
Wang et al, 2020^14^	A systematic review and metanalysis of studies assessing cardiovascular events during major Football tournaments like the European Championship World Cup; English Football Tournament; Australian Football League; World Cup Qualifications; besides Rugby World Cup, and Super Bowl taking part between 1980 and 2011	The pooled RR was 1.17 (95% CI 1.01–1.36) for non-fatal acute cardiovascular events and 1.03 (95% CI 1.00–1.05) for vascular mortality
Wilbert-Lampen et al 2008^15^	FIFA World Cup, 2006 in Germany	The incidence of cardiac emergencies was 2.66 times that during the control period (95% CI 2.33 to 3.04; P<0.001).
Carroll et al, 2002^16^	FIFA World Cup, 1998, in England	The admissions due to myocardial infarction increased by 25% on the day England lost to Argentina
Witte et al, 2000^17^	European football championship in 1996	The mortality from coronary heart disease increased among men on the day of the match when the Dutch football team was eliminated from the European football championship (relative risk 1.51, 95% confidence interval 1.08 to 2.09)
Keller et al, 2021^18^	FIFA World Cup, 2014, in Brazil	The number of hospital admissions caused by myocardial infarction was 3.7% higher in Germany during the FIFA World Cup 2014 than during the same 31-day period in 2015
Puche, 2022^19^	Spanish League competitions during 2018–2020	An increase in the number of admissions due to acute coronary syndrome by 30% in males with cardiac risk factors on the days the local team lost.
Fijalkowski et al, 2013^21^	European Cup, 2012	A case study of Takotsubo cardiomyopathy in a 56-year-old man with no past cardiovascular history
Elamin et al, 2021^22^	English Football League, 2018	A case study of Takotsubo cardiomyopathy in a 79-year-old Sheffield United fan

Other major sporting events can also trigger cardiac incidents. One study showed a significant increase in cardiac deaths following the Rams’ dramatic loss in the 1980 Super Bowl (the annual final playoff game of the National American Football League).^23^ In New Zealand, investigating the cardiac-related admissions during four tournaments of the Rugby World Cup (RWC) showed a doubling of the pooled heart failure admissions and a 20% increase in pooled acute coronary syndromes admissions.^24^ Similarly, cardiac incidents have been reported among fans of Hockey, and baseball games^25^,^26^.

### Pathophysiology of Stress-Induced Cardiac Events with a Particular Focus on Football Games

**Figure 1:**
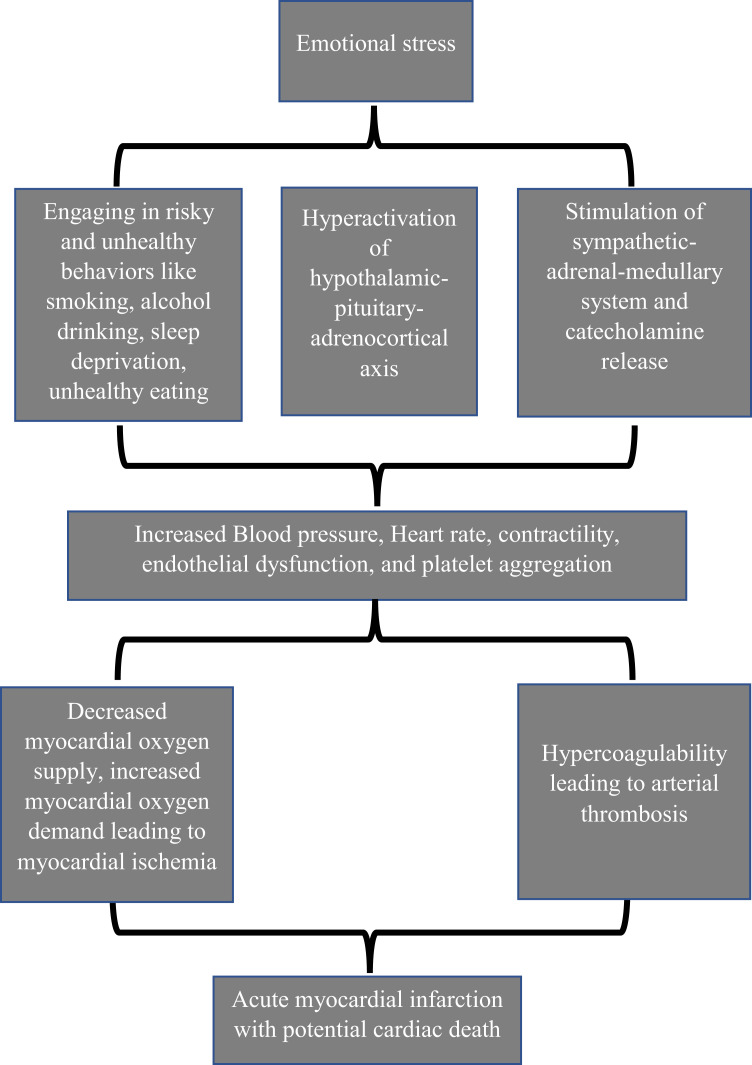
Pathophysiology of stress-induced cardiac events.

Acute mental stress hyperactivates the hypothalamic-pituitary-adrenocortical axis and the sympathetic-adrenal-medullary system.^27^ The increased sympathetic tone and catecholamine levels while watching an important football match are likely to adversely influence the cardiovascular system and precipitate cardiac events, particularly in individuals with predisposing factors for CVD.^28^ The sympathetic hyperstimulation and catecholamine rush elevate the heart rate, blood pressure, and myocardial contractility resulting in a relative reduction in the oxygen supply to the heart muscle.^29^ Moreover, the elevated level of catecholamines can precipitate arrhythmias and platelet aggregations^29^ (Figure 1). Takotsubo cardiomyopathy is to a great extent similar to acute myocardial infarction but in the absence of obstructive epicardial coronary artery disease and characterized by transient left ventricular systolic and diastolic dysfunction, electrocardiographic features, and myocardial enzyme elevation.^30^ Elevated levels of circulating plasma catecholamines and their metabolites are among some of the mechanisms proposed for the development of Takotsubo syndrome.^31^

The literature has shown that football fans who are strongly fused or bonded with their teams are more likely to experience higher concentrations of the “fight or flight” cortisol hormone which is an important mediator of the stress response, which sometimes can reach dangerous levels.^32^,^33^ Identity fusion is a strong form of group alignment in which there is a synergistic activation of personal and group identities to produce a visceral sense of “oneness” with one’s team. Evidence that was gathered at field laboratories during the 2014 FIFA World Cup in Brazil showed that participants who experienced the greatest stress response system activation with higher cortisol levels over the course of a World Cup match also tended to be the most fused^32^. A study that investigated the levels of testosterone and cortisol among Spanish fans watching the finals between Spain and the Netherlands in the 2010 FIFA World Cup Football showed higher levels of these hormones when watching the match than on a control day. Moreover, the heightened cortisol secretion during the match was more among men than women, younger, and stronger fans (those who reported the highest levels of fandom).^34^ Additionally, the behaviors usually associated with watching important matches such as smoking, overeating, excessive drinking, and sleep deprivation could further contribute to adverse cardiac outcomes.^8^,^27^,^29^

### Strategies for Tackling Stress-Induced Cardiac Events During the FIFA World Cup 2022

During major sporting events, early measures must be taken to control stress and subsequent cardiac events among participants. Messages that focus on reducing stress, educating people about its dangerous consequences, and publicizing stress-coping strategies must be included as an integral part of any planned health-promoting campaign. These messages must target all people including fans and football players themselves. People should be encouraged to cut down on risky and unhealthy behaviors such as smoking, eating unhealthy food choices, overeating, excessive drinking, prolonged sitting, and sedentary behaviors. Qatar will continue implementing FIFA’s tobacco-free policy during the World Cup as it did during the FIFA Club World Cup Qatar 2019™ to ensure an environment free of tobacco and protect the health of non-smoking spectators and the workforce from passive or secondhand smoke. Examples of measures taken during the FIFA Club World Cup Qatar 2019™ were prohibiting the lighters and matches from stadiums, establishing Outdoor Designated Smoking Areas (ODSA), installing “No Smoking/No Vaping” signage throughout the stadium perimeter, and deploying stewards and volunteers across all matches to help in the implementation of the tobacco-free Policy and to guide smokers to ODSA.^35^ Other risk factors for CVD including HTN, DM, and dyslipidemia should be controlled, and people should be encouraged to continue complying with their medication and follow up closely with their physicians. People need to be advised about the importance of getting adequate sleep and avoiding sleep deprivation during the period of the tournament. It is important to warn people at risk about the potential cardiac symptoms that warrant seeking urgent medical attention. There should be appropriate precautions (like defibrillators and personnel trained in their use) in place when triggering cardiac events might occur, like inside the stadiums and in fan zones. Moreover, standard procedures for getting symptomatic individuals to the nearest health facility must be ensured.^36^ Patients with preexisting mental health conditions like depression and anxiety should be advised to communicate with their psychiatrists to receive the necessary counseling and care to prevent any relapses. Establishing a mental helpline staffed by a team of mental health professionals who can provide the needed psychosocial support for callers during the World Cup is of utmost importance. Working closely with elite football players from all the participating teams to help spread and reinforce health-related messages on how to keep the mind and body healthy and how to reduce stress, particularly among fans can be instrumental to prevent the psychosocial consequences of such major sporting events.

In collaboration with the World Health Organization (WHO), Qatar launched a three-year joint project titled “Healthy 2022 World Cup - Creating Legacy for Sport and Health” to place the promotion of healthy lives, health security, and physical and mental well-being at the heart of the World Cup and to make this event as the healthiest sport event possible.^37^ Paying a great deal of attention to the mental health of people before, during, and after such events is needed especially since the unprecedented crisis of COVID-19 had a negative psychological impact on many people around the world.

## Conclusion

Major sporting events can trigger acute cardiac events among both competitors and spectators, and the FIFA World Cup is a prime historical example of this. Recovering from the negative repercussions the COVID-19 pandemic has had on the mental health of people requires collaboration, solidarity, determination, and continuous efforts to help people protect their minds from further challenges. Carrying the measures implemented during the pandemic forward into the World Cup like the mental helpline can be beneficial. Being the most widely viewed and followed single sporting event in the world, the FIFA World Cup is an opportunity for all countries in the world, not just the hosting country to advance health awareness messages with a focus on mental health and stress management to their people. While watching football matches during the World Cup, people should always remember that a Healthy Mind is needed for A healthy Heart and by the end “it is just a game”.
